# Unexpected systemic sclerosis in a patient with atopic dermatitis receiving dupilumab: a novel case report

**DOI:** 10.1093/skinhd/vzaf100

**Published:** 2026-01-09

**Authors:** Ela Gazal, Saffet Burak Başak, Yasemin Yuyucu Karabulut, Ümit Türsen

**Affiliations:** Department of Dermatology, Mersin University Hospital, Mersin, Türkiye; Department of Dermatology, Mersin University Hospital, Mersin, Türkiye; Department of Pathology, Mersin University Hospital, Mersin, Türkiye; Department of Dermatology, Mersin University Hospital, Mersin, Türkiye

## Abstract

Dupilumab, an interleukin (IL)-4 receptor alpha antagonist, is widely used in the treatment of atopic dermatitis and has shown potential benefit in certain fibrosing skin conditions. While blockade of IL-4 and IL-13 is generally considered to inhibit fibrosis, emerging reports of localized sclerosing dermatoses paradoxically arising during dupilumab therapy suggest a more complex immunological effect. We report a 37-year-old woman with the first case of systemic sclerosis developing in a patient with atopic dermatitis treated with dupilumab. After 2 years of treatment, she presented with new-onset dyspnoea, Raynaud phenomenon and digital puffiness. Serological tests revealed positive antinuclear and anticentromere antibodies; thoracic computed tomography showed interstitial lung disease. The modified Rodnan skin score was 4, and a skin biopsy from the fingertip demonstrated compact hyperkeratosis, irregular acanthosis and fibrosis extending to the superficial dermis. The patient met the American College of Rheumatology/European Alliance of Associations for Rheumatology 2013 classification criteria for systemic sclerosis, with a total score of 10. Dupilumab was discontinued, and systemic treatments targeting vascular symptoms and pulmonary involvement were initiated, resulting in clinical improvement. This case highlights a significant potential paradoxical reaction associated with cytokine-targeted biologic therapy. It underscores the importance of monitoring for fibrotic and systemic symptoms even when using agents presumed to exert antifibrotic effects.

## Teaser

Dupilumab is widely used for atopic dermatitis and is believed to reduce fibrosis by blocking interleukin (IL)-4/IL-13. We present the first reported case of systemic sclerosis emerging during dupilumab therapy. A 37-year-old woman developed Raynaud’s phenomenon, digital puffiness and interstitial lung disease 2 years after treatment began. Diagnostic investigations confirmed systemic sclerosis with an American College of Rheumatology/European Alliance of Associations for Rheumatology 2013 score of 10. This unexpected outcome highlights the complex immunologic effects of cytokine blockade and the need for vigilance regarding fibrotic symptoms in treated patients. The case raises important questions about immune shifts and paradoxical responses in targeted biologic therapies.

What is already known about this topic?

- Interleukin (IL)-4 and IL-13 blockade with dupilumab is generally considered protective in fibrotic conditions due to its anti-T-helper 2 effects.

What does this study add?

- This is the first reported case of systemic sclerosis emerging during dupilumab treatment, challenging the assumed antifibrotic role of IL-4/IL-13 inhibition.

Systemic sclerosis (SSc) is a chronic autoimmune connective tissue disorder marked by microvascular injury, immune dysregulation and progressive fibrosis of the skin and internal organs. Current evidence implicates a multifaceted interplay of genetic, environmental and immune factors in its pathogenesis. The disease often follows a sequence beginning with vascular abnormalities, progressing to inflammation and culminating in fibrosis. Among the key cytokines implicated in this process are interleukin (IL)-4 and IL-13, which have been shown to stimulate fibroblast activity and collagen deposition while suppressing extracellular matrix degradation. These findings have positioned the IL-4/IL-13 axis as a potential therapeutic target for halting or reversing fibrosis in SSc.^1^

Dupilumab, a monoclonal antibody that inhibits IL-4Rα and blocks both IL-4 and IL-13 signalling, has been shown to be effective in treating atopic dermatitis and has exhibited potential in various fibrosing skin conditions, such as keloids and morphoea.^2^ Based on this mechanism, some researchers have hypothesized that dupilumab may also benefit patients with SSc by mitigating type 2-driven fibrotic pathways.^1^ However, emerging case reports have paradoxically linked dupilumab to the development of localized sclerotic conditions, such as morphoea, suggesting that the immunological consequences of IL-4/IL-13 blockade may be more complex than initially believed.^3–5^

## Case report

**Figure 1:**
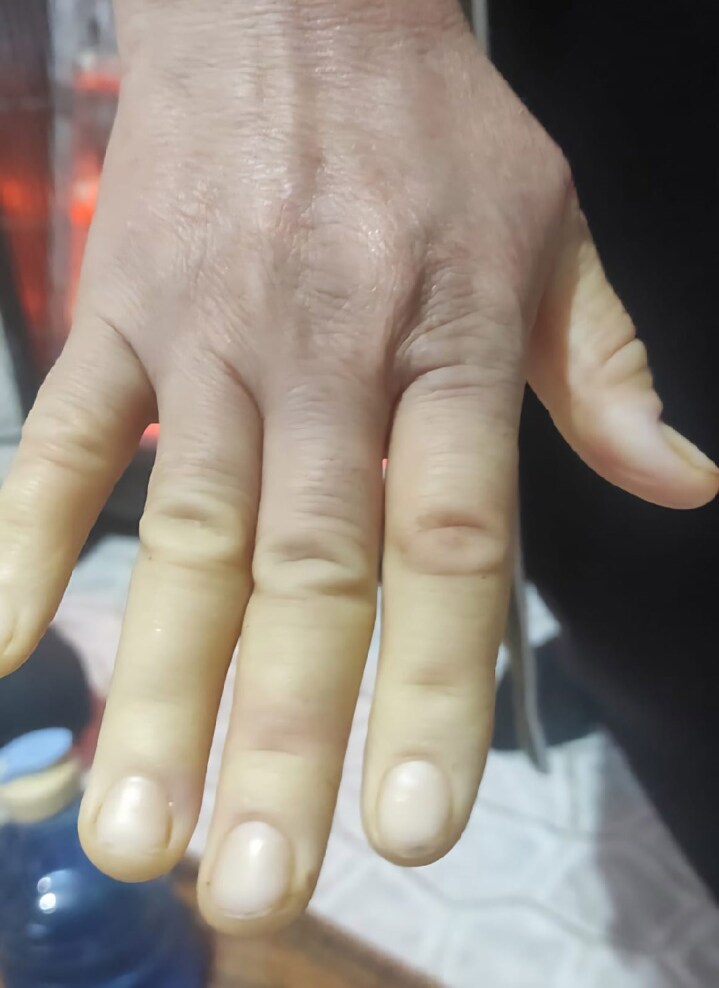
Raynaud phenomenon in the patient. The photograph shows the characteristic blanching of the fingers, consistent with the clinical presentation of Raynaud phenomenon.

**Figure 2:**
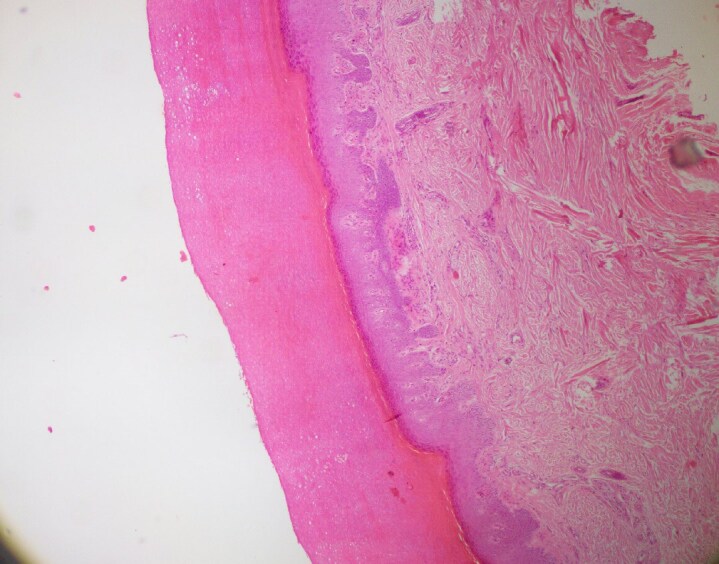
Compact hyperkeratosis, irregular acanthosis and thickened dermal collagen extending toward the epidermis (haematoxylin and eosin ×40).

**Figure 3:**
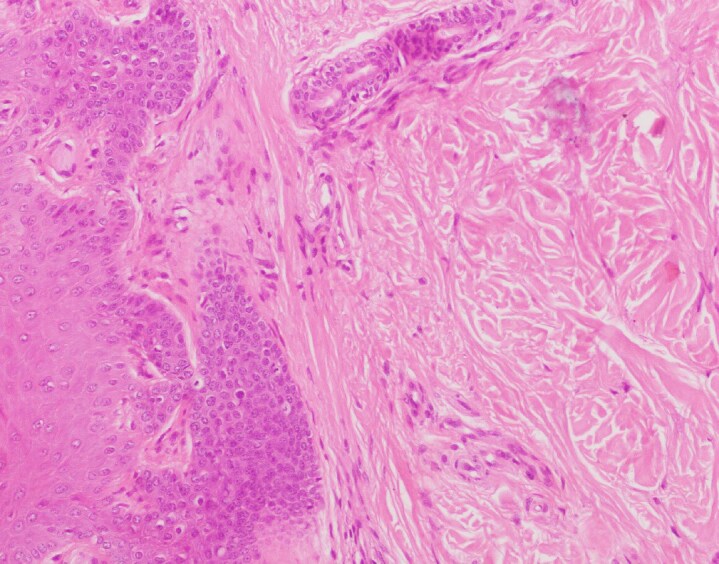
Sparse perivascular lymphocytes and histiocytes within thickened dermal collagen (haematoxylin and eosin ×200).

A 37-year-old woman with a history of atopic dermatitis unresponsive to corticosteroids and ciclosporin was started on dupilumab therapy. Two years into treatment, she developed new-onset exertional dyspnoea, chest pain, Raynaud phenomenon (Figure 1) and digital puffiness. Thoracic computed tomography revealed patchy ground-glass opacities in the lungs. Serology was positive for antinuclear and anticentromere antibodies. Nailfold capillaroscopy, echocardiography and Doppler ultrasonography were unremarkable. On physical examination, no thickening or shortening of the lingual frenulum was observed. However, limited skin involvement of the fingers and hands was noted, with a modified Rodnan skin score (mRSS) of 4. A skin biopsy from the fingertip demonstrated compact hyperkeratosis, irregular acantosis and thickened dermal collagen extending toward the epidermis, with sparse perivascular lymphocytes and histiocytes, in keeping with fibrotizing dermatitis (Figures 2, 3). She met the American College of Rheumatology/European Alliance of Associations for Rheumatology 2013 classification criteria for SSc, with a total score of 10 (Table 1), exceeding the threshold of 9 required for classification, based on clinical, radiological, histological and serological findings. Due to the temporal relationship between dupilumab initiation and symptom onset, dupilumab was discontinued. Topical treatments for atopic dermatitis were resumed, and systemic therapies including nifedipine, aspirin, cilostazol and inhaled bronchodilators were introduced to manage SSc-related symptoms. At follow-up, improvement was observed in Raynaud phenomenon and dyspnoea; however, no change was noted in mRSS.

**Table 1:** American College of Rheumatology/European Alliance of Associations for Rheumatology 2013 classification criteria for systemic sclerosis (SSc) applied to our patient (a total score ≥9 is required for classification)

Criteria	Definition	Present in our patient	Score
Skin thickening of the fingers	Puffy fingers	Yes	2
	Sclerodactyly (distal to MCPs)	No	
Fingertip lesions	Digital tip ulcers or pitting scars	No	
Telangiectasia	Visible dilated capillaries	Possible mild facial telangiectasia-like findings	0^a^
Abnormal nailfold capillaries	On capillaroscopy	No	
Pulmonary arterial hypertension and/or ILD	ILD on HRCT or PAH by RHC	Yes (ground-glass opacities on CT)	2
Raynaud phenomenon	History or observed	Yes	3
SSc-related autoantibodies	ACA, anti-topoisomerase I or anti-RNA polymerase III	Yes (ANA, ACA positive)	3
Total score			10

ACA, anticentromere antibody; ANA, antinuclear antibody; CT, computed tomography; HRCT, high-resolution computed tomography; ILD, interstitial lung disease; MCPs, metacarpophalangeal joints; PAH, pulmonary arterial hypertension; RHC, right heart catheterization. ^a^Possible facial telangiectasia was noted but not scored due to lack of formal documentation.

## Discussion

The paradoxical emergence of SSc during dupilumab therapy raises important questions about the nuanced role of immune modulation in fibrosing disorders. It is well established that type 2 cytokines promote fibroblast activation, transforming growth factor-β production and extracellular matrix deposition.^6,7^ In contrast, type 1 cytokines such as interferon-γ may counteract fibrosis by suppressing collagen production. Nevertheless, IL-4 and IL-13 are only part of a broader immune network. Indeed, recent studies suggest that T helper (Th) 1, Th17 and Th22 cells also infiltrate SSc skin lesions, and may act in both proinflammatory and regulatory capacities.^6^ In addition, elevated levels of group 1 innate lymphoid cells, which share features with Th1 cells, have been documented in the blood of patients with SSc.^8^ These findings suggest that shifting immune responses – either through disease progression or therapeutic intervention – could influence fibrosis in unexpected ways.

Similar paradoxical fibrosing reactions have been described in patients receiving dupilumab. To date, only a few cases of morphoea or localized scleroderma developing during dupilumab treatment have been reported in the literature, with variable latencies from drug initiation to onset of fibrosis. González Fernández *et al*. recently described a case of generalized morphoea developing 6 months after dupilumab was started for nodular prurigo, supporting the possibility that IL-4/IL-13 blockade can disrupt immune balance and favour fibrosis.^3^ Other reports include two paediatric cases of morphoea developing after 34 and 5 months of dupilumab exposure, respectively, in which an exaggerated Th1/Th17 response was hypothesized to initiate the early inflammatory phase.^4^ In another case, morphoea occurred after 8 months of treatment, and it was proposed that IL-4 blockade may upregulate the IL-4δ2 splice variant, skewing the immune response toward Th1 activation and subsequent fibrosis.^5^ Together, these cases suggest that paradoxical fibrosing reactions can occur both early and late in dupilumab therapy and may arise through different immune mechanisms, emphasizing the need for continued monitoring even during long-term treatment. Although speculative, these theories emphasize the potential for immune axis disruption to trigger paradoxical fibrosis, especially in individuals with predisposing genetic or immunological factors. Importantly, our patient represents, to our knowledge, the first reported instance of SSc developing during dupilumab treatment, extending the spectrum of fibrosing conditions potentially linked to IL-4/IL-13 pathway inhibition.

The clinical implications are twofold. Firstly, clinicians should maintain vigilance for early signs of fibrosis in patients receiving dupilumab, especially if new systemic or vascular symptoms arise. Secondly, the immune network driving fibrosis in SSc is likely more intricate than a linear Th2-driven process. This complexity underscores the need for deeper exploration into the dynamic interplay between immune pathways in fibrotic diseases. Future studies should aim to delineate the immunological profiles of patients before and after targeted cytokine blockade, with attention to paradoxical responses and long-term effects.

In conclusion, while dupilumab represents a promising therapeutic agent for Th2-mediated conditions, its role in fibrotic diseases remains uncertain. This case highlights a significant potential adverse effect and calls for further research into the immunological shifts triggered by targeted cytokine inhibition. Understanding these mechanisms will be crucial in refining treatment strategies for SSc and other fibrotic disorders.

### Conflicts of interest

The authors declare no conflicts of interest.

## Data Availability

All data underlying this article are included within the article and its figures.
